# Clinical Metagenomic Next-Generation Sequencing for Diagnosis of Secondary Glaucoma in Patients With Cytomegalovirus-Induced Corneal Endotheliitis

**DOI:** 10.3389/fmicb.2022.940818

**Published:** 2022-07-05

**Authors:** Wei Wu, Hua Jiang, Ying Zhang, Yang Zhou, Guannan Bai, Lingwei Shen, Hongwei Zhou, Xiangjun Chen, Lidan Hu

**Affiliations:** ^1^Eye Center, The Second Affiliated Hospital, School of Medicine, Zhejiang University, Hangzhou, China; ^2^Zhejiang Provincial Key Lab of Ophthalmology, The Second Affiliated Hospital, School of Medicine, Zhejiang University, Hangzhou, China; ^3^Department of Otolaryngology, The Second Affiliated Hospital, Zhejiang University School of Medicine, Hangzhou, China; ^4^Institute of Translational Medicine, Zhejiang University School of Medicine, Hangzhou, China; ^5^BGI PathoGenesis Pharmaceutical Technology Co., Ltd., Hangzhou, China; ^6^The Children’s Hospital, National Clinical Research Center for Child Health, Zhejiang University School of Medicine, Hangzhou, China; ^7^Department of Clinical Laboratory, The Second Affiliated Hospital Zhejiang University School of Medicine, Hangzhou, China

**Keywords:** secondary glaucoma, cytomegalovirus, metagenomic next-generation sequencing, herpes simplex virus, varicella-zoster virus

## Abstract

Glaucoma is the second leading cause of blindness globally. Growing scientific evidence indicated that inflammation of the trabecular meshwork induced by corneal endotheliitis could lead to secondary glaucoma. Cytomegalovirus (CMV) has been identified as the most common herpes virus in corneal endotheliitis patients. Early detection is critical in preventing endothelial cell loss, and patient management should vary based on different pathological factors. However, routine culture and real-time polymerase chain reaction (qPCR) have difficult in distinguishing whether CMV, Varicella Zoster Virus (VZV) or Herpes Simplex Virus (HSV) causes endothiliitis. This may result in inappropriate treatment, which may prolong or aggravate the status of disease. We compared the sensitivity and specificity of qPCR and Metagenomic Next-Generation Sequencing (mNGS) in the aqueous humor of patients with suspected CMV endotheliitis in this study. Our results showed that four out of 11 (36.4%) of our patients were positive for CMV by qPCR, whereas mNGS had a 100% detection rate of CMV. Our findings implied that mNGS could be a useful diagnostic tool for CMV-induced endotheliitis.

## Introduction

Glaucoma is characterized by loss of retinal ganglion cells and visual field constriction, which is the second leading cause of blindness globally (Cook and Foster, 2012; Baudouin et al., 2021). Glaucoma can be categorized as primary and secondary glaucoma (Foster et al., 2002; McMonnies, 2017). Compared to primary glaucoma, secondary glaucoma may be caused by eye injury, inflammation, certain drugs, cataract, or diabetes, etc. (McMonnies, 2017). Based on different pathological factors, effective precision management should be applied to improve patients’ health-related quality of life and to preserve visual function. However, so many cases of glaucoma remain undiagnosed or insufficiently treated, especially the inflammation (Khodadoust and Attarzadeh, 1982). Glaucoma is associated with toxic inflammatory factors, thereby leading to cell death and disease progression (Baudouin et al., 2021). Several inflammatory processes could result in corneal edema, and the most common status would progress to endotheliitis. Endotheliitis is defined as inflammation of the corneal endothelium and is thought to be related to viral infection, especially in association with the cytomegalovirus (CMV) (Koizumi et al., 2015), herpes simplex virus (HSV) (Ohashi et al., 1991), and varicella-zoster virus (VZV) (Inoue and Ohashi, 2013). Moreover, it has been demonstrated that CMV is the most common herpes virus infected corneal endotheliitis patients, with clinical features such as coin-shaped KP and elevated intraocular pressure (IOP) (Chee et al., 2007; Koizumi et al., 2015; Kim et al., 2018). The choice of the treatment regimen for endotheliitis depends on the underlying etiology of the disease process. However, some inadequate treatments, which include antiviral medication like acyclovir, have been applied in most cases of endotheliitis, even to those patients who were not detected with the virus. Such unwarranted treatments would, beyond doubt, have a poor prognosis, especially in patients with elevated IOP (Ohashi et al., 1985). Therefore, exploring a quick and accurate method to detect the infection pathogens in patients with endotheliitis is imperative for early diagnosis and subsequent proper treatment. This might ultimately prevent the progression of the disease.

Pathogen identification can provide solid laboratory evidence for making correct clinical diagnosis and for guiding clinical treatment for secondary glaucoma, accordingly. Several detection methods have been used in clinical diagnosis, including routine culture, real-time Polymerase chain reaction (qPCR), and enzyme-linked immunosorbent assay (ELISA). qPCR has been regarded as an accurate diagnostic approach to detect viral DNA in the aqueous humor (Kandori et al., 2010; Inoue, 2014). And it has been applied in detecting CMV-induced corneal endotheliitis (Nakagawa et al., 2021). Immunoassays, often indicated as IgM and IgG seropositivity via the ELISA, have also proven to be effective in diagnosing these viral etiologies (Koizumi et al., 2015). However, limited aqueous humor or contamination of the sample restricted the detection of various viruses through these traditional methods (Ohashi et al., 1985; Chronopoulos et al., 2016). Therefore, it is imperative to establish a more effective and sensitive approach to detect infection pathogens for diagnose.

Metagenomic next-generation sequencing (mNGS) technique comprises high-throughput sequencing and bioinformatics that offer detection of pathogens (Bertelli and Greub, 2013; Chiu and Miller, 2019). By using this technique, pathogens can be detected from different types of samples, such as cerebrospinal fluid (CSF) (Mai et al., 2017; Wilson et al., 2017; Fan et al., 2018), respiratory secretions (Langelier et al., 2018), and blood (Greninger and Naccache, 2019). In addition to detection of the pathogen such as viruses, fungi, bacteria, mNGS has the potential to be a useful tool for retrieving antimicrobial gene information (Miao et al., 2018; Dulanto Chiang and Dekker, 2020). This would give researchers more information for better understanding the interaction between hosts and microorganisms. Here, we presented a series of patients suffered from secondary glaucoma with CMV-induced corneal endotheliitis that were diagnosed using mNGS. Based on the definite diagnosis by mNGS, clinicians adopted combination therapy (oral GCV and topical GCV gel) for these CMV-positive patients, which proved be effective treatment. The earlier the better to prevent the corneal endothelium damage, which hence require early accurate diagnosis. We urgently aimed to elucidate the application of mNGS in CMV-induced corneal endotheliitis and improve the cure rate of secondary glaucoma.

## Materials and Methods

### Case Series

From January 2019 to May 2022, thirteen patients, including 11 patients with secondary glaucoma and clinically suspected CMV endotheliitis and two negative control patients with Posner-Schlossman syndrome (PSS), were enrolled in the eye center of the Second Affiliated Hospital of Zhejiang University School of Medicine (SAHZU). This study was carried out in accordance with the principles of the Helsinki Declaration, and the study protocol was approved by the SAHZU’s institutional review/ethics boards (number: IR2019001040). The following criteria were met by all of the patients with suspected CMV endotheliitis in this study: (1) coin-shaped keratic precipitates (KPs) and (2) increased intraocular pressure (IOP). Patients with the following conditions were excluded: (1) presence of vitreous or retinal inflammation, and (2) presence of corneal endothelial changes for a known cause other than anterior uveitis. Patients were subjected to thorough examinations, including slit lamp microscope, intraocular pressure, optometry, anterior segment photography, endothelium cell count, gonioscope and confocal microscopy. Seven aqueous humor samples from the pathogenic findings, and treatment and outcomes data were recorded in the hospital information system.

### Aqueous Humor Collection and Processing

All aqueous humor sample collections were performed using a standard sterilization procedure. Undiluted aqueous humor samples (200 μL from one eye) were obtained from the patients via anterior chamber paracentesis. The samples were flash-frozen and kept at 80°C. 1.5 mL microcentrifuge tube with 200 μL sample and 250 μL 0.5 mm glass bead (BioSpec Products, OK, United States) were attached to a horizontal platform on a vortex mixer (Vortex-Genie2 vortex mixer, Scientific Industries, United States) and agitated vigorously at 2,800–3,200 rpm for 30 min. Then 7.2 μL lysozyme (Tiangen Biotech, Beijing, China) was added for wall-breaking reaction. 0.3 mL sample was separated into a new 1.5 mL microcentrifuge tube and DNA was extracted using the TIANamp Micro DNA Kit (DP316, Tiangen Biotech, Beijing, China) according to the manufacturer’s recommendation.

### DNA Library Construction and Data Analysis

DNA libraries were constructed through DNA-fragmentation, end-repair, adapter-ligation and PCR amplification using the MGIEasy DNA Library Prep Kit (MGI, Wuhan, China). Agilent 2100 (Agilent Technologies, CA, United States) and Qubit 3.0 (Thermo Fisher Scientific, United States) platform were used for library quality control. Quality qualified libraries (200–300 bp of average length, > 2 ng/μL) were pooled, DNA Nanoball (DNB) was made and sequenced by BGISEQ-50/MGISEQ-2000 platform.

### Bioinformatic Analysis

High-quality sequencing data was generated by removing low-quality reads. The RefSeq data was downloaded on Jan 2022. The quality check of Fastq data was performed by an in-house software with three quality controlling steps: (1) removal of reads less than 35 bp; (2) removal of reads which had more than 30% bases of pared 33 score less than 5; (3) removal of reads containing more than 10 unspecified bases. Followed by computational subtraction of human host sequences mapped to the human reference genome (hg19) using Burrows-Wheeler Alignment. Non-specific sequences that could be aligned to multiple candidate genomes within different genus were removed by the in-house software and wouldn’t go through the BWA alignment. We used Burrows-Wheeler Alignment Tool for the alignment to the reference database (parameter: bwa mem −k 19 −t 8 −Y −h 10,000). The remaining data by removal of low-complexity reads were classified by simultaneously aligning to Pathogens meta-genomics Database (PMDB), consisting of bacteria, fungi, viruses and parasites. The classification reference databases were downloaded from NCBI.^1^ RefSeq contains 4,945 whole genome sequence of viral taxa, 6,350 bacterial genomes or scaffolds, 1,064 fungi related to human infection, and 234 parasites associated with human diseases. Generally speaking, if the number of CMV reads in sample was more than three, the sample should be considered to define as “CMV-positive.”

### Real-Time PCR Assays

CMV DNA copies were detected by the diagnostic kit for qPCR of Human CMV (Sansure Biotech, China) following the manufacturer’s protocol. The process of the CMV DNA amplification and product analysis was performed by an ABI StepOne Plus Sequence Detection System (Applied Biosystems, Foster City, CA, United States). PCR conditions included a pre-denaturation step at 94°C for 5 min, followed by 40 cycles denaturation at 94°C for 10 s, annealing at 63°C for 45 s. In our laboratory, less than 1.0 × 10^2^ copies/mL of CMV DNA was considered negative. The positive control, negative control, and four calibrations were included in each reaction.

### Statistical Analysis

All data are expressed as the mean ± standard error of the mean (SEM) of three independent experiments.

## Results

### Clinical Findings and Pathogen Detection

#### Demographic Feature

**FIGURE 1:**
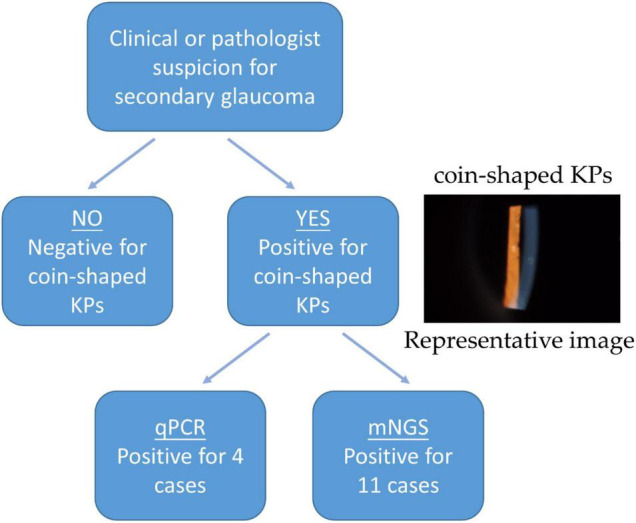
The proposed procedure utilizing qPCR and mNGS analysis on the aqueous humor of CMV-infected patients.

The secondary glaucoma caused by CMV corneal endotheliitis is caused by cytomegalovirus and could cause irreversible vision loss. Clinical manifestations are characteristic coin-shaped KP, increased intraocular pressure, and normal immune function, accompanied by corneal edema and loss of corneal endothelium. In addition, two negative control patients were Posner-Schlossman syndrome (PSS). These 11 patients were enrolled in this study. During the follow-up schedules, 11 patients were diagnosed with secondary glaucoma, along with coin-shaped KPs (Figure 1). The basic clinical manifestations and demographic data of patients are listed in Table 1. The mean age for the patients was 45.18 ± 16.12, calculated from the range between 18 and 69 years. Seven of the patients in this study were male. There were no reports of HIV infection or previous reception of corneal transplantation in all patients. However, all patients had been treated for ocular hypertension with endotheliitis at other ophthalmology clinics and prescribed with topical steroid and glaucoma medications. Six of the 11 patients had received 0.15% topical ganciclovir (GCV) gel therapy.

**TABLE 1:** The demographic data and clinical manifestations of patients at baseline.

Case	Age (years)	Gender	Coin-shaped KPs^a^	Corneal edema^a^	AC inflammation^a^	BCVA (decimal fraction)	IOP (mmHg) (No. of glauma medications)	Prior treatment	ECD (cells/mm^2^)	
									Affected	Unaffected
1	69	M	1 +	+	+	0.8	34.5 (3)	G	1,410	2,557
2	42	M	1 +	_	_	1.0	39 (3)	T	2,820	2,874
3	45	F	1 +	+	+	0.8	24 (2)	T	758	2,453
4	29	F	2 +	+	+	0.6	32 (3 + Tb)	G	2,729	2,688
5	49	F	1 +	+	+	0.6	21 (3 + Tb)	G	1,254	2,886
6	39	M	2 +	+	+	0.4	37 (3 + Tb)	G	2,781	2,774
7	18	M	1 +	_	_	1.0	20 (2)	T	2,534	3,056
8	60	M	2 +	+	+	0.1	57.5 (3 + Tb)	T	1,493	2,934
9	53	M	2 +	+	+	0.1	29.3(3)	G	1,522	2,731
10	28	F	2 +	_	_	1.0	25 (1)	G	2,817	2,667
11	65	M	2 +	+	+	0.8	60 (2)	T	2,899	3,045
C1	33	M	_	_	_	0.6	29 (3)	T	2,311	2,776
C2	29	F	_	_	_	1.0	23 (1)	T	2,708	3,011

*^a^Severity of Coin-shaped KPs, Corneal edema and AC inflammation were graded as no findings, 1 + (mild), 2 + (moderate) and 3 + (severe).*

*KPs, keratic precipitates; AC, anterior chamber; BCVA, best-corrected visual acuity; IOP, intraocular pressure; ECD, endothelial cell density; M, male; F, female; Tb, acetazolamide tablet; GCV, ganciclovir; DXM, Dexamethasone. G, GCV gel + Topical DXM; T, Topical DXM; C, control.*

The clinical evaluation in our department found coin-shaped KPs in all 11 eyes (100%), corneal edema in eight eyes (72.7%), and mild anterior chamber inflammation in eight eyes (72.7%). The mean IOPs and best-corrected visual acuity (BCVA) of 11 affected eyes were 34.48 ± 13.54 mmHg and 0.65 ± 0.33, respectively. The average of endothelial cell density (ECD) in 11 affected eyes was 2092.45 ± 800.83 cells/mm^2^, which was lower than that in the contralateral unaffected eyes (2787.73 ± 192.32 cells/mm^2^).

After CMV infection was confirmed, all patients were received oral GCV and topical GCV gel. Seven patients in the early stages of the CMV-induced corneal endotheliitis were well controlled after 4 weeks combination therapy, including coin-shaped KPs disappeared and IOP returned to normal. Whereas, the four remaining patients did not receive ideal treatment results, who had secondary glaucoma lasted for several years and obvious optic nerve damage in the late disease stage. Subsequently, these four patients underwent trabeculectomy and IOP decreased to normal levels.

#### Pathogen Detection

**FIGURE 2:**
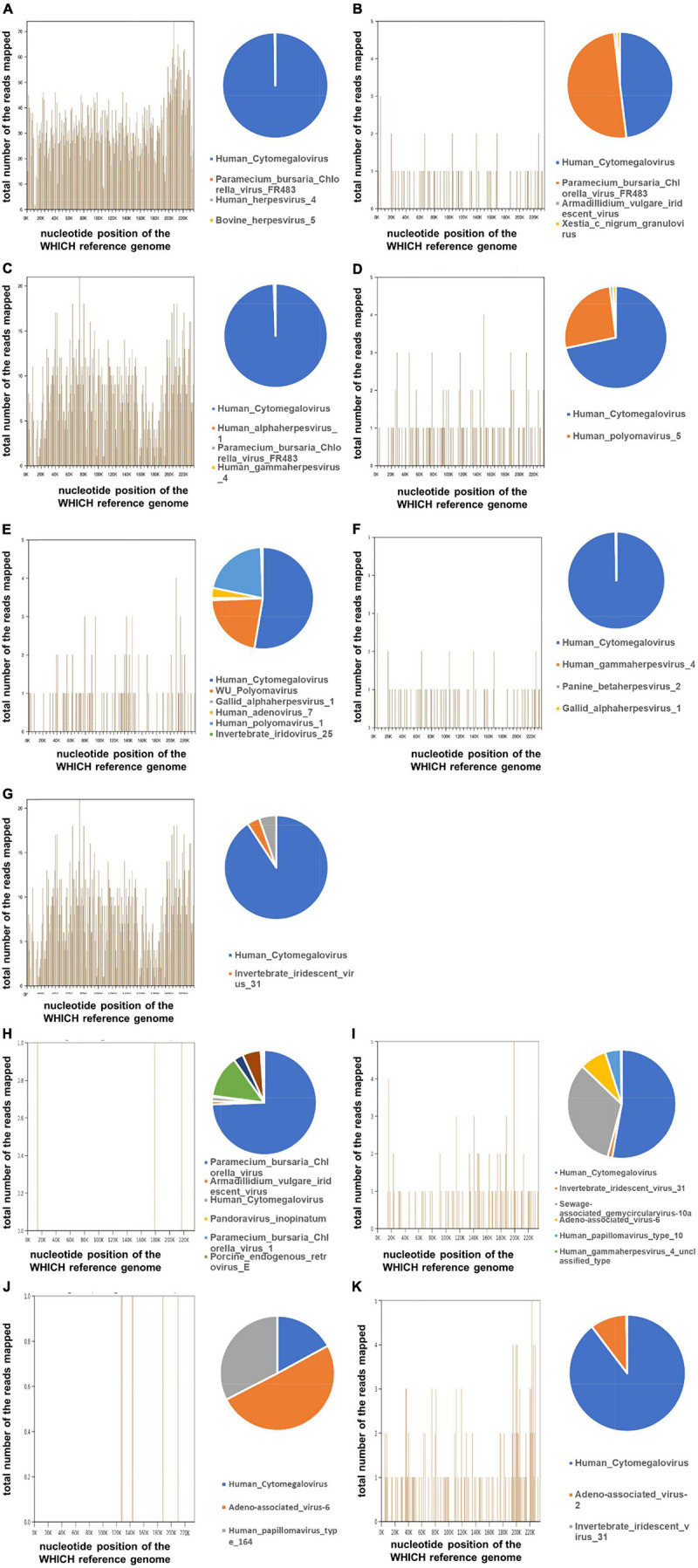
Detection of the Human Cytomegalovirus (CMV) by metagenomic Next-generation sequencing (mNGS) on the aqueous humor from 11 glaucoma patients. The total DNA from aqueous humor samples from the eyes of the patient with suspected CMV infection was sequenced by BGISEQ-50/MGISEQ-2000 platform. The reads were mapped against human reference genome (hg19) using Burrows-Wheeler Alignment. From different samples, a different number of reads matching to CMV were recovered. The 3 number of the reads were considered as the “CMV-positive.” Genome coverage of detected CMV sequences and the relative abundance of detected viruses in the 11 patients **(A–K)**.

In our case, we have tested the aqueous humor from all the 11 patients, who were clinically considered with secondary glaucoma due to CMV-induced corneal endodermatitis, at different sampling times and onset times. Specifically, seven aqueous humor samples were collected at early onset (within 6 months) and four aqueous humor samples were collected in the later (mean 3.5 years). In our laboratory, less than 1.0 × 10^2^ copies/mL of CMV DNA was considered negative. Under this standard, four out of 11 (36.4%) patients were positive for CMV-DNA by qPCR analysis, whereas seven were negative (Table 2). Three of the seven samples collected at the onset were tested as positive, while one of the four samples collected during the late disease course was interpreted as positive. The mean cytomegalovirus DNA copy numbers were 5.74 × 10^4^ copies/mL. To test our therapy, all the aqueous humor of the 11 affected eyes underwent mNGS analysis and all the patients were diagnosed of potential CMV infections (Table 3). Table 3 also indicated that many other reads were detected in samples, including bacteria, viruses, fungi, parasites, and unmatched sequences. Raw CMV reads were identified ranging from 3 to 355,961, with genomic coverage ranging from 0.06 to 94.58%. The detailed results of mNGS are shown in Figure 2. In brief, CMV remained the dominant species in all the analyzed samples.

**TABLE 2:** Viral load in CMV infected aqueous humor.

Case	Viral load (copies/mL)
1	1.31 × 10^5^
2	8.75 × 10^4^
3	Neg
4	Neg
5	Neg
6	8.2 × 10^2^
7	Neg
8	Neg
9	Neg
10	Neg
11	1.60 × 10^2^

**TABLE 3:** The mNGS of aqueous humor of 11 affected eyes.

Case	Total non-human reads	CMV reads	Std. CMV reads^a^	CMV Genome coverage (%)	Total number of human reads (%)	Total number of non-human reads (%)	Other human viruses genome coverage (%)
Pt-1	822,752	7,654	21,356	74.57	99.77	0.23	0.03
Pt-2	1675,660	64	243	1.36	48.07	51.93	0
Pt-3	3,912,516	1,892	3,128	31.44	99.53	0.47	0.11
Pt-4	3,049,196	118	362	2.43	98.08	1.92	0.92
Pt-5	3,173,565	109	215	2.29	76.97	23.03	0.90
Pt-6	14,738,023	355,961	261,493	94.58	99.75	0.25	3.07
Pt-7	6,776,006	25	32	0.53	9.76	90.24	0
Pt-8	840,564	3	4	0.06	6.89	93.11	0
Pt-9	2,123,584	335	158	1.63	57.76	42.24	0
Pt-10	2,721,083	17	6	0.10	49.66	50.34	0
Pt-11	921,902	446	242	2.75	89.88	10.12	0.12

*mNGS, metagenomic next-generation sequencing; RPM, species-specific reads per million of total reads.*

*^a^CMV reads per 20 M of total reads, 200 μL sample volume, DNA Library type.*

## Discussion

Corneal endotheliitis was first described by Khodadoust and Attarzadeh (1982), (Paul, 1988). CMV-induced corneal endotheliitis was often characterized by linear or circularly organized (often termed coin-shaped) KPs, corneal edema, and mild anterior chamber inflammation (Koizumi et al., 2006). However, not all patients with CMV endotheliitis have such clinical characteristics (Chiang et al., 2011). Koizumi et al. (2015) reported that coin-shaped lesions were observed in 70.6% of the eyes with CMV endotheliitis and linear KPs were noted in 8.3% eyes. Although the clinical features of CMV endotheliitis enable us to make a diagnosis for some patients, more comprehensive and accurate approaches are still necessary for the more exact detection of the virus. Currently, there is no consensus on the management of CMV endotheliitis (Wong et al., 2021). Thus, early diagnosis is crucial for preventing endothelial cell loss, which could ultimately lead to corneal decompensation.

In this study, we collected all 11 patients who appeared with coin-shaped KPs. Recently, the performance of qPCR on aqueous humor was evaluated on patients suffering from secondary glaucoma and typical signs of corneal endotheliitis (Harper et al., 2009; Nakagawa et al., 2021). Harper et al. (2009) assessed the potential application of PCR on intraocular fluid in viral infectious cases. The sensitivity and specificity of PCR were 80.9 and 97.4%, respectively, in a total of 133 patients. The PCR had some limitations due to the small volume of aqueous humor, including low efficiency and sensitivity to detect various types of viruses. PCR results were positive in four out of 11 (36.4%) of our patients. However, mNGS had a 100% detection rate of CMV in samples, which was significantly higher than the PCR evaluation.

Similar to the results that were reported by De Groot-Mijnes et al. (2006) real-time PCR missed nearly half of the infectious cases in intraocular fluids. Furthermore, Davies et al. (2005) also reported false-negative results for viral infection detection in CSF. The researchers discovered that positive PCR results were more likely in the early stages of viral infection (de Boer et al., 1996; Davies et al., 2005; De Groot-Mijnes et al., 2006). The most plausible explanation was that the pathogen load was reduced to below the detection limit later in the infection, or that viral particles were released slowly (De Groot-Mijnes et al., 2006). However, it is difficult to determine when the patients came to detect after viral infection, which may limit the use of PCR in detecting the microbes. These reasons brought the conclusion that PCR was not a satisfactory tool for clinical applications. To find more effective and sensitive detection methods for CMV-induced corneal endotheliitis diagnosis, we applied the mNGS technique in elven aqueous humor samples from secondary glaucoma patients for CMV detection for the first time in this study.

Previous research demonstrated that mNGS is a promising universal pathogen detection method for infectious diseases such as respiratory tract infections (Langelier et al., 2018), bloodstream infections (Greninger and Naccache, 2019), bone (Chen et al., 2020), joint infections (Cai et al., 2020) and ocular infections (Doan et al., 2016). The mNGS can identify bacteria, fungi, parasites, and viruses in intraocular fluid samples for ocular diseases. Doan et al. (2016) identified the chronic rubella virus in minute volumes of intraocular fluid samples, thereby emphasizing the eye’s role as a long-term pathogen reservoir, which was later confirmed by qPCR. Furthermore, similar studies that used mNGS for detecting various pathogens such as CMV, HHV-6, and HSV-2 revealed that metagenomic DNA sequencing was highly concordant with pathogen-directed PCRs (Doan et al., 2017).

Secondary glaucoma caused by CMV-induced corneal endotheliitis is more common in immunocompromised individuals or those who have previously undergone corneal transplantation (Koizumi et al., 2015). The reason could be that CMV is an opportunistic organism that can reactivate in the presence of systemic or local immunosuppressive agents (Doan et al., 2016). CMV-related endotheliitis, on the other hand, occurs in immunocompetent individuals, in contrast to CMV infections in general (Inoue, 2014). According to our case series, all the patients were immunocompetent and denied using any immunosuppressive medication. The pathogenic mechanism of CMV endotheliitis is yet to be identified. Zheng et al. (2000) demonstrated that anterior chamber-associated immune deviation may play a pivotal role in the progression of viral corneal endotheliitis.

CMV-induced corneal endotheliitis is difficult to distinguish from other viral corneal endotheliitis forms, such as the ones caused by VZV or HSV. The inappropriate treatment, which commonly involves the use of acyclovir, may prolong or worsen the condition. In our cases, after CMV endotheliitis confirmed by mNGS, all patients received oral GCV and topical GCV gel. Seven patients in the early stages of the CMV-induced corneal endotheliitis were well controlled. Whereas, four patients had to undergo trabeculectomy. To conclusion, the early GCV treatment may be more effective for patients with CMV-induced corneal endotheliitis. It is consistent with what previous researchers reported (Koizumi et al., 2015). The poor response to GCV therapy may be related to a long-term viral infection that causes inflammation and damages the filtration function of the trabecular meshwork cells (Koizumi et al., 2008; Choi et al., 2017). Meanwhile, the mNGS results indicated that some patients may suffer from mix infection, not only limit by CMV, which may lead the treatment more challenging. Apart from bacteria, fungi, viruses, and parasites, many unmapped non-human reads were also detected in our samples. The reason of recognized these reads included: (1) Sequences with no match any genomes in the database; (2) sequences with high homology between species; (3) sequences with low specificity and accurately. This emphasizes the importance of developing a new method for early detection and preventive and management measures of CMV endotheliitis.

The relatively small sample size of our study was a limitation. Because the selected cases had endotheliitis characterized by coin-shaped KPs which is rare, the local COVID-19 outbreaks disturbed our data collection. We made our best effort to collect 11 cases. However, the study design was scientific by comparing to the golden standard qPCR method, so the present study can provide solid evidence to support that mNGS can be used as a promising technique to diagnose endotheliitis caused by CMV compared to the qPCR. Taken for granted, mNGS, as a high-throughput sequencing technology, required more costs and time costs compared to qPCR. Meanwhile, since mNGS unbiased sequence the genome in the sample, it detects commensal and opportunistic microbes simultaneously with the potential pathogen. Thus, it should be combined with clinical information to get a more practical and objective evaluation.

## Conclusion

In conclusion, we investigated the use of mNGS on the aqueous humor to detect CMV in secondary glaucoma patients with a clinical suspicion of CMV endotheliitis. Through the limited samples detection, we could see the mNGS has a bright future in detect the CMV endotheliitis compared with qPCR, particularly when clinical features are atypical or routine laboratory results contradict clinical findings.

## Data Availability Statement

The data presented in this study are deposited in the NCBI repository, accession number: PRJNA844122.

## Ethics Statement

The studies involving human participants were reviewed and approved by the Eye Center of The Second Affiliated Hospital of Zhejiang University School of Medicine’s Institutional Review/Ethics Boards (number: IR2020001040). The patients/participants provided their written informed consent to participate in this study. Written informed consent was obtained from the individual(s) for the publication of any potentially identifiable images or data included in this article.

## Author Contributions

WW, HJ, and LH: conception and design of study. WW, HJ, and YiZ: data collection. XC, YaZ, GB, LS, and HZ: analysis and interpretation of results. WW, XC, and LH: drafting the manuscript. All authors reviewed the results and approved the final version of the manuscript.

## Conflict of Interest

YaZ was employed by BGI PathoGenesis Pharmaceutical Technology Co., Ltd. The remaining authors declare that the research was conducted in the absence of any commercial or financial relationships that could be construed as a potential conflict of interest.

## Publisher’s Note

All claims expressed in this article are solely those of the authors and do not necessarily represent those of their affiliated organizations, or those of the publisher, the editors and the reviewers. Any product that may be evaluated in this article, or claim that may be made by its manufacturer, is not guaranteed or endorsed by the publisher.

## Footnotes

1
https://ftp.ncbi.nlm.nih.gov/genomes/

## Abbreviations

CMV: Cytomegalovirus
CSF: Cerebrospinal fluid
ECD: Endothelial cell density
ELISA: Enzyme-linked immunosorbent assay
GCV: Ganciclovir
HSV: Herpes Simplex Virus
IOP: Intraocular pressure
mNGS: Metagenomic next-generation sequencing
PCR: Polymerase chain reaction
VZV: Varicella Zoster Virus.
